# Enzymatic Analysis of Yeast Cell Wall-Resident GAPDH and Its Secretion

**DOI:** 10.1128/mSphere.01027-20

**Published:** 2020-12-16

**Authors:** Michael J. Cohen, Brianne Philippe, Peter N. Lipke

**Affiliations:** a Biology Department, Brooklyn College of the City University of New York, Brooklyn, New York, USA; b The Graduate Center of the City University of New York, New York, New York, USA; University of Texas Health Science Center

**Keywords:** cell wall, disulfide reduction, membrane integrity, enzyme assay, spheroplast

## Abstract

In yeast, many proteins are found in both the cytoplasmic and extracellular compartments, and consequently it can be difficult to distinguish nonconventional secretion from cellular leakage. Therefore, we monitored the extracellular glyceraldehyde-3-phosphate dehydrogenase (GAPDH) activity of intact cells as a specific marker for nonconventional secretion. Extracellular GAPDH activity was proportional to the number of cells assayed, increased with incubation time, and was dependent on added substrates. Preincubation of intact cells with 100 μM dithiothreitol increased the reaction rate, consistent with increased access of the enzyme after reduction of cell wall disulfide cross-links. Such treatment did not increase cell permeability to propidium iodide, in contrast to effects of higher concentrations of reducing agents. An amine-specific membrane-impermeant biotinylation reagent specifically inactivated extracellular GAPDH. The enzyme was secreted again after a 30- to 60-min lag following the inactivation, and there was no concomitant increase in propidium iodide staining. There were about 4 × 10^4^ active GAPDH molecules per cell at steady state, and secretion studies showed replenishment to that level 1 h after inactivation. These results establish conditions for specific quantitative assays of cell wall proteins in the absence of cytoplasmic leakage and for subsequent quantification of secretion rates in intact cells.

**IMPORTANCE** Eukaryotic cells secrete many proteins, including many proteins that do not follow the classical secretion pathway. Among these, the glycolytic enzyme glyceraldehyde-3-phosphate dehydrogenase (GAPDH) is unexpectedly found in the walls of yeasts and other fungi and in extracellular space in mammalian cell cultures. It is difficult to quantify extracellular GAPDH, because leakage of just a little of the very large amount of cytoplasmic enzyme can invalidate the determinations. We used enzymatic assays of intact cells while also maintaining membrane integrity. The results lead to estimates of the amount of extracellular enzyme and its rate of secretion to the wall in intact cells. Therefore, enzyme assays under controlled conditions can be used to investigate nonconventional secretion more generally.

## Abstract (precis)

Eukaryotic cells secrete many proteins, including many proteins that do not follow the classical secretion pathway. Among these, the glycolytic enzyme glyceraldehyde-3-phosphate dehydrogenase (GAPDH) is unexpectedly found in the walls of yeasts and other fungi and in extracellular space in mammalian cell cultures.

## INTRODUCTION

The glycolytic enzyme glyceraldehyde-3-phosphate dehydrogenase (GAPDH) is unexpectedly found in the walls of Saccharomyces cerevisiae, Candida albicans, and Paracoccidioides brasiliensis (1–8). The enzyme is also secreted from mammalian cells in culture (9–11). Like many glycolytic proteins, GAPDH is a moonlighting protein with additional roles both within the cell (12, 13) and externally; in the C. albicans cell wall GAPDH binds fibronectin (14), and in S. cerevisiae its secreted form is cleaved into antimicrobial peptides (15, 16). Recent cell wall proteomics work has shown cell wall localization of GAPDH in C. albicans and non-albicans species (17, 18). The protein is present in walls, and in many cases its concentration is increased after growth in media that mimics mammalian conditions. Thus, GAPDH is a common wall marker in pathogenic yeasts and may be important in host-pathogen interactions.

All three isoforms of GAPDH, encoded by *TDH1*, *TDH2*, and *TDH3*, are enzymatically active in S. cerevisiae walls (1), but cell surface quantities and pathways leading to secretion remain elusive. Mass spectrometry of protease-treated cell walls or of intact cells generates peptides from enolase, alcohol dehydrogenase, and GAPDH as well as cytosolic chaperones such as Ssa1 and Ssa2 in both S. cerevisiae (4) and C. albicans (6, 19). Thus, GAPDH is prototypical of many unconventionally secreted proteins (20), as defined by their presence in the extracellular compartment despite their lack of canonical secretion signal peptides.

S. cerevisiae can be engineered to display and anchor enzymes on the cell wall for biofuel production (21), bioremediation (22), or library screening (23). The cell walls consist of polysaccharides, including β1,3 glucans, β1,6 glucans, chitin, and a large number of proteins. These cell wall-resident proteins cross-link the saccharides, act as adhesins, regulate metabolic activities, and perform other functions (24–26). Most of these proteins are secreted through the conventional secretion signal-dependent pathway that processes the proteins through the endoplasmic reticulum (ER) and Golgi membrane (27, 28). This pathway was famously elucidated through a combination of enzymology and genetic screens (28). Temperature-sensitive S. cerevisiae secretory mutants were generated, and at nonpermissive temperatures they showed defects in invertase and acid phosphatase secretion (29).

Yeasts have also been used to study unconventional protein secretion of proteins which lack a signal peptide. S. cerevisiae expressing the mammalian protein Galectin-1 could secrete it without using its classical secretory system (30), much as the protein behaves in mammalian cells (31). Mutational studies in S. cerevisiae identified an Acb1 secretory mechanism that requires autophagy, Golgi proteins, and endosome components (32, 33). The chitinase Cts1 from Ustilago maydis was used to study a novel form of unconventional protein secretion at budding sites (34). Yeast species are also used to characterize unconventional secretion into extracellular vesicles (20, 35–37). Thus, yeast is now a classic model for study of secretory pathways in general.

We are interested in studying unconventional secretion of proteins such as GAPDH using an enzymology approach. However, since GAPDH is abundant in the cytosol, there is a critical need to obtain cell wall extracts while avoiding cytosolic contamination. Therefore, we describe procedures for quantitative assay of extracellular GAPDH and techniques for its extraction without contamination by cytosolic enzymes.

## RESULTS

Because enzyme assays of cell wall-resident proteins have the potential to generate quantitative and kinetic information about secretion, we characterized cell surface GAPDH activity.

### GAPDH activity in the wall of intact S. cerevisiae.

**FIG 1:**
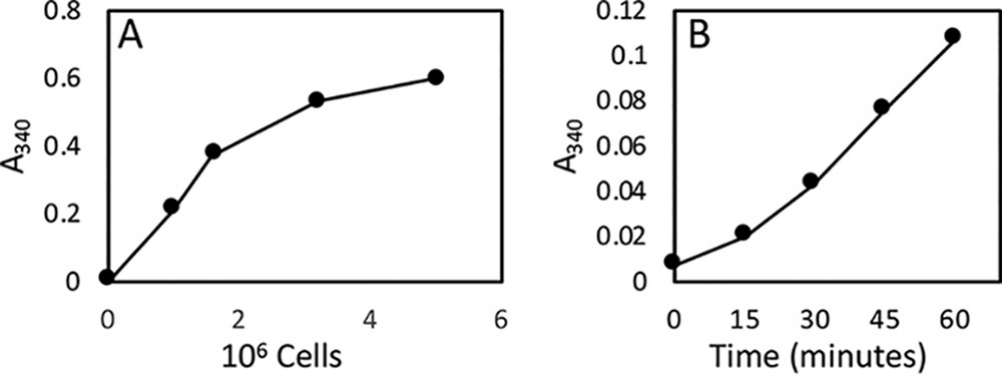
Enzymological characteristics of cell wall GAPDH assays. (A) Dependence on cell number in a 30-min assay. (B) Time course of the assay with 5 × 10^5^ cells.

We verified that GAPDH is enzymatically active in the wall of S. cerevisiae strain BY4743 by resuspending cells in 1 mM NAD, 1 mM glyceraldehyde-3-phosphate, 100 μM dithiothreitol (DTT), and triethanolamine phosphate (TEA) buffer (pH 8.6) in a 200-μl reaction mixture, using methods similar to those of Delgado et al. (1). However, we extended their results by establishing the wall-associated activity on a per-cell basis. We suspended different concentrations of S. cerevisiae in GAPDH substrates for 30 min and then measured NADH production. Yeast cells were pelleted by centrifugation, and NADH was measured as the *A*_340_. NADH production was linear with cell number up to 1.5 × 10^6^ per 200-μl reaction mixture (Fig. 1A). Therefore, subsequent experiments used a maximum of 1 × 10^6^ cells in 200 μl, with a majority of trials using 5 × 10^5^ cells. The reaction rate was linear between 30 and 60 min but showed a lag before that time (Fig. 1B). The origin of the lag is addressed in the next section.

Other enzymological controls were as expected. Specifically, as negative controls, there was no measurable activity in the absence of either substrate NAD^+^ or glyceraldehyde-3-phosphate. These data show that all assayed NAD^+^ reductase activity was due to GAPDH, and there was no endogenous activity due to leakage of either substrate from the cytoplasm. The absorbance spectrum of the product matched NADH, and the optimum reaction pH was 8.6, consistent with known GAPDH properties (38). This pH value suggests that cell surface GAPDH is not enzymatically active during yeast growth, which normally occurs under acidic conditions.

### Cell surface GAPDH activity increases during assays.

**FIG 2:**
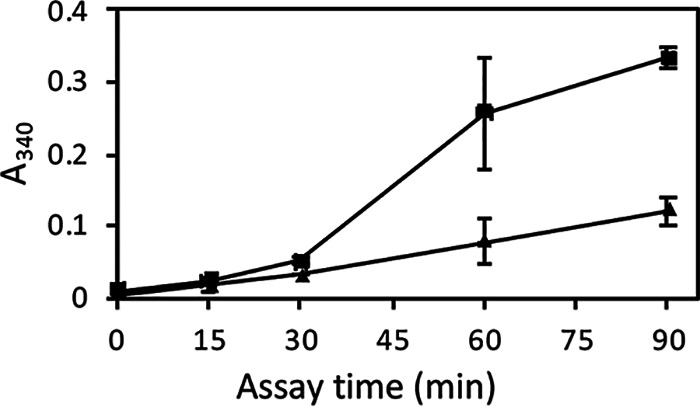
Cell surface GAPDH activity on the presence and absence of DTT. GAPDH assays in the absence (▲) and presence (▪) of 100 μM DTT over 90 min.

In 60- and 90-min assays, the rate of NADH production increased (Fig. 2). This increase emphasized the lag time apparent in Fig. 1B. This result suggested that either GAPDH was accumulating at the surface or that more extracellular GAPDH became active during the extended incubation in assay buffer.

GAPDH can be partially oxidized *in vivo*, reducing its activity by 10%, so standard assays contain DTT to keep the enzyme reduced (39). Our assay buffer contained 100 μM DTT, as in Delgado et al. (1, 2). However, DTT can also break disulfide bonds in the wall and increase cell wall porosity (5, 40), exposing more enzyme to substrates (41–43). To test whether DTT was facilitating increased cell wall GAPDH activity, we assayed activity in the presence or absence of 100 μM DTT. The rate of NADH production was greater in the presence of DTT than without it, and the rate of increase was maximal between 30 and 60 min of incubation (Fig. 2). This finding was confirmed in a preincubation experiment. Cells were preincubated in assay buffer in the absence of substrate and in the presence or absence of DTT (100 μM). Substrates were then added and enzyme activity monitored in standard 30-min assays. Preincubation in DTT increased GAPDH activity at the cell surface in a subsequent GAPDH assay. Therefore, DTT treatment either increased the fraction of surface GAPDH that was enzymatically active, promoted surface accumulation of the enzyme, made the wall more accessible to reagents, or some combination of the above.

### GAPDH on the surface can be attenuated with a membrane-impermeant covalent modifier.

**FIG 3:**
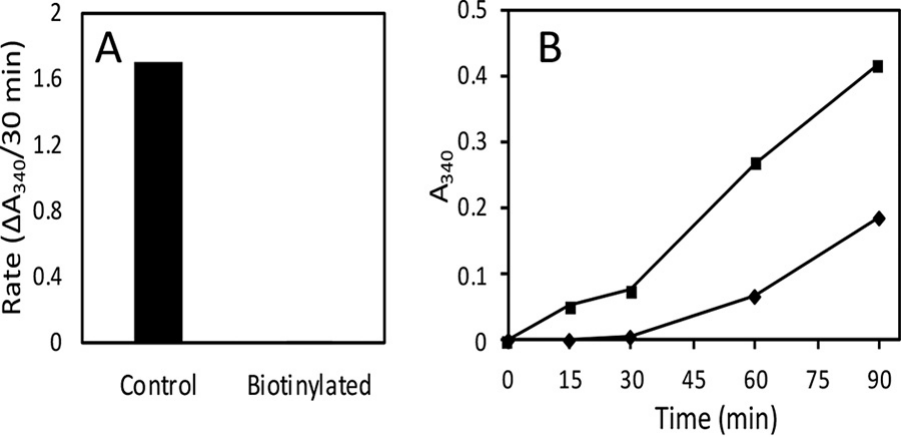
Effect of sulfo-NHS-LC biotin on GAPDH activity. (A) Cytosolic lysate biotinylated with 1 mg/ml sulfo-NHS biotin has low GAPDH activity. Lysate was biotinylated, 10 μl of 1:5 dilutions of lysate was loaded onto a microtiter plate, 90 μl of substrates was added, and the *A*_340_ was monitored over 30 min. (B) Yeast grown to an OD of 0.7 were biotinylated (♦) for 1 h in PBS, pH 7, with 1 mg/ml sulfo-NHS-LC biotin or PBS (▪) and then suspended in TEA buffer containing GAPDH substrates for 15, 30, 60, and 90 min. Supernatant (180 μl) was loaded onto a microplate, and the *A*_340_ was determined. Points are averages from 2 samples.

To distinguish between GAPDH already in the wall and newly secreted GAPDH, we took advantage of a membrane-impermeant modifier to deactivate cell wall-associated GAPDH. Attempts to label and extract cell surface GAPDH led to the observation that the biotinylation reagent sulfosuccinimidyl 6-(biotinamido)-hexanoate (sulfo-NHS-LC biotin) decreased GAPDH activity dramatically, both in cytoplasmic extracts and for the enzyme assayed on the surface of intact cells (Fig. 3). Because sulfo-NHS-LC biotin is membrane impermeant (44) and will only react with proteins external to the plasma membrane, it can specifically deactivate cell wall GAPDH and leave cytosolic GAPDH unaffected. We treated intact cells with sulfo-NHS-LC biotin and then washed the cells to remove the remaining sulfo-NHS-LC biotin, resuspended in assay buffer, and assayed for 15 to 90 min. Unlike the control untreated cells, biotinylated yeast did not have detectable GAPDH activity on their surfaces for the first 30 min (Fig. 3). However, biotinylated yeast showed increasing surface GAPDH between 30 and 90 min (Fig. 3B). This result was consistent with GAPDH being released to the surface from a cellular pool inaccessible to sulfo-NHS-LC biotin, presumably in the cytoplasm. However, the rate of increase of cell surface activity was less than that of the cells not treated with biotinylation reagent. Therefore, about half of the increase shown in Fig. 2 and 3 may be due to secretion of active enzyme to the cell surface during the incubation, but some of the increase may represent more activity of the resident assayable cell surface enzyme, presumably due to the wall permeabilization by DTT.

Sulfo-NHS-LC biotin covalently modifies primary amines and consequently may have an effect on all surface proteins and cause secondary effects. Therefore, we measured invertase activity in yeast that were either biotinylated or incubated in phosphate-buffered saline (PBS) to see if all surface proteins become dysfunctional upon biotinylation. Invertase activity was unaffected by sulfo-NHS-LC biotin (see Fig. S1 in the supplemental material). Therefore, biotinylation inactivated GAPDH, but not invertase, consistent with specific modification of GAPDH rather than global perturbation of either wall structure or metabolic activities.

10.1128/mSphere.01027-20.1 FIG S1 Invertase activity of intact cells with and without treatment with sulfo-NHS-LC biotin. Yeast were grown in YPGal medium, washed, left untreated or treated with sulfo-NHS-LC biotin, incubated with or without sucrose, and reducing sugar determined. Download FIG S1, TIF file, 1.6 MB. Copyright © 2020 Cohen et al. 2020 Cohen et al. This content is distributed under the terms of the Creative Commons Attribution 4.0 International license.

### Enzyme assay conditions do not permeabilize the plasma membrane.

**FIG 4:**
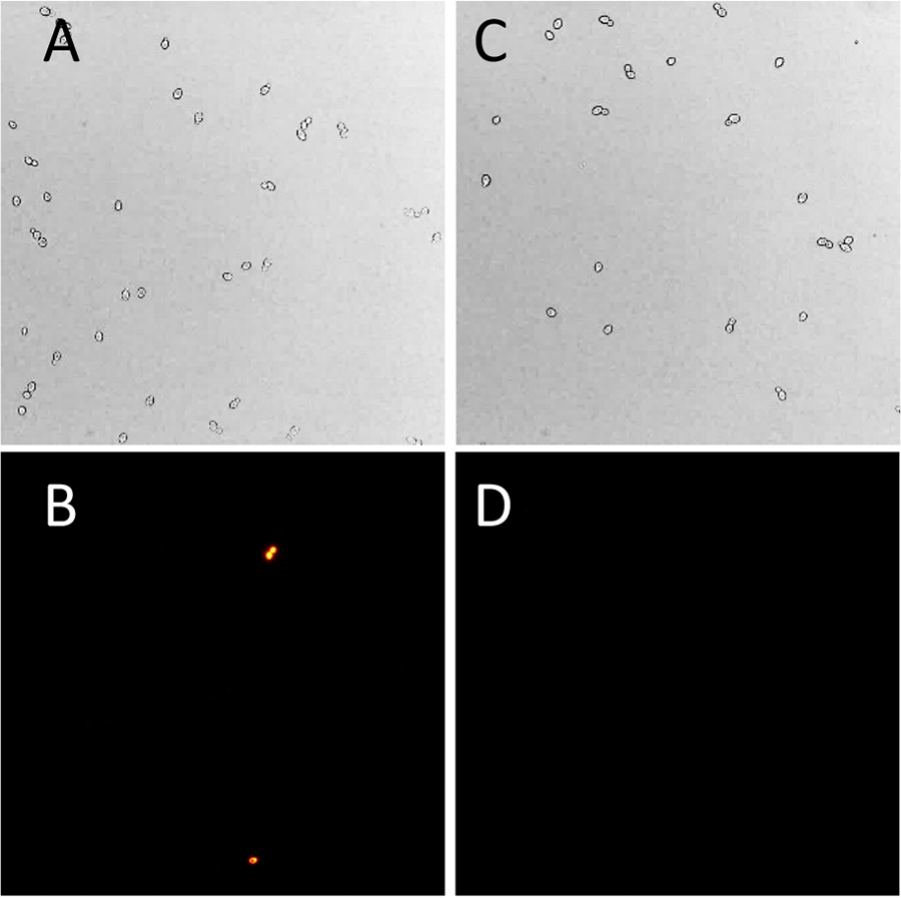
Propidium iodide staining of S. cerevisiae after a GAPDH assay. S. cerevisiae suspended in TEA buffer, pH 8.6, before (A and B) or after (C and D) assays for GAPDH surface activity. Top, bright-field image; bottom, propidium iodide epifluorescence in the same field.

In assays of cell wall enzymes, it is important that the plasma membrane is not permeabilized, so that all of the assayed activity derives from extracellular enzyme. Therefore, we compared propidium iodide (PI) staining before and after assaying S. cerevisiae for GAPDH surface activity. There was no visible increase in the fraction of PI-positive yeast after assaying yeast for cell wall GAPDH (Fig. 4). Therefore, the increase in GAPDH activity seen after 100 μM DTT treatments and recovered after biotinylation was likely due to enzymes externalized by controlled biological processes and unlikely to be caused by plasma membrane leakage.

**FIG 5:**
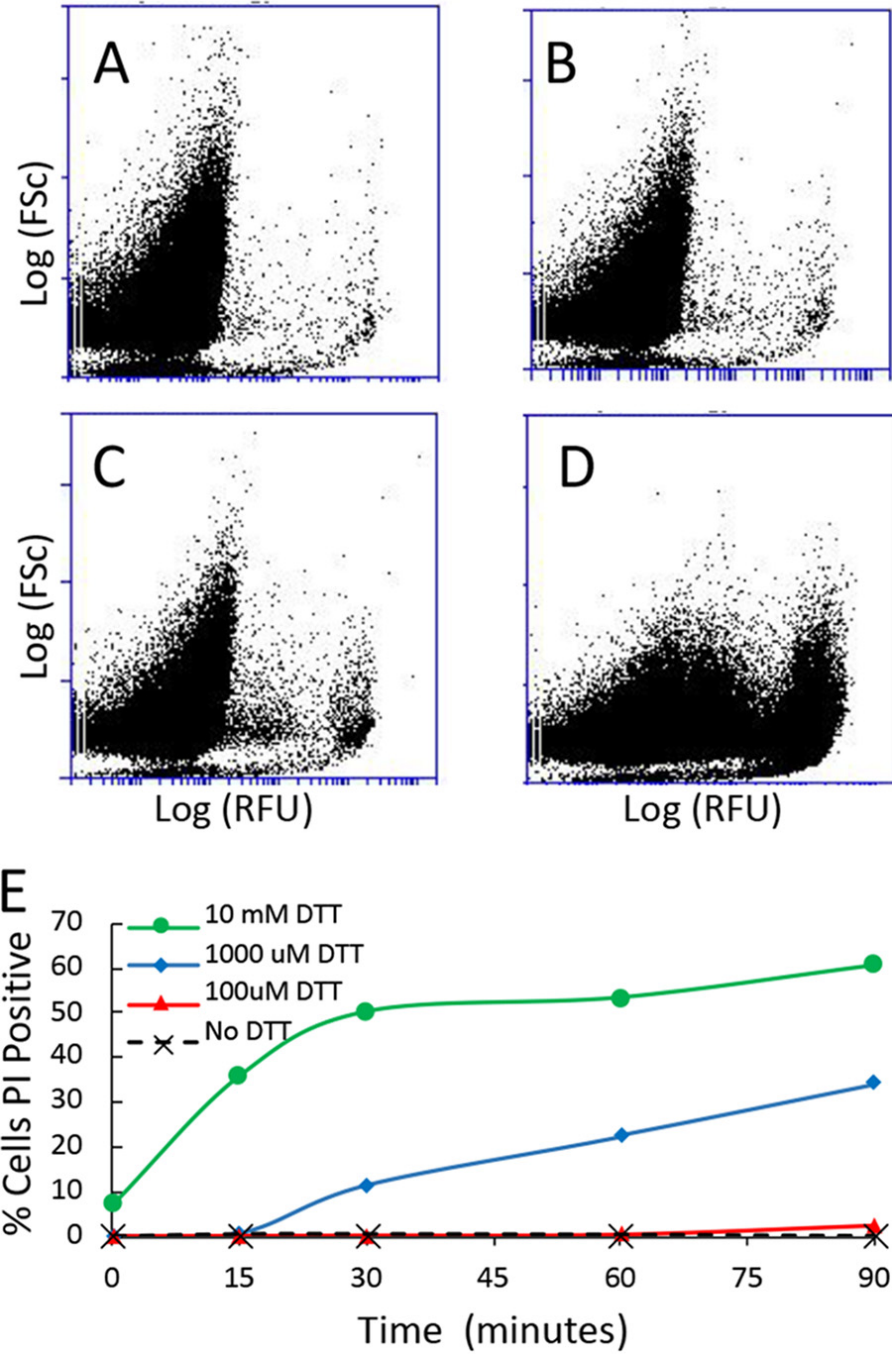
Flow cytometry of effect of DTT on propidium iodide staining of S. cerevisiae cells. (A to D) Propidium iodide fluorescence after a 60-min incubation at 30°C in TEA buffer pH 8.6. (A) No DTT; (B) 100 μM DTT; (C) 1 mM DTT; (D) 10 mM DTT. (E) Percentage of cells that were PI positive. DTT concentrations were 0 (X), 100 μM (red triangles), 1 mM (blue diamonds), and 10 mM (green circles).

We also found that high concentrations of reducing agents can permeabilize the plasma membrane (discussed later), so we wanted to ensure DTT concentrations used in whole-cell assays for GAPDH on the surface were not permeabilizing the plasma membrane. We incubated S. cerevisiae in a TEA buffer at pH 8.6, at 30°C in different concentrations of DTT, and monitored PI fluorescence over time with flow cytometry. The results demonstrated that a high concentration of DTT (1 mM or higher) permeabilized the plasma membrane, but 100 μM did not cause a significant amount of cells to become PI positive compared to a nontreated control group, and this was consistent over 90 min (Fig. 5). Other reducing agents, including β-mercaptoethanol (5 to 14 mM) or tris(2-carboxyethyl)phosphine (TCEP) (5 mM), also increased PI staining of cells, implying permeabilization of the plasma membrane (Fig. S2). Therefore, we utilized 100 μM DTT for all subsequent assays.

10.1128/mSphere.01027-20.2 FIG S2 Exposing yeast to reducing agents can release cell wall enzymes but also permeabilizes cells. (A) GAPDH activity in supernatant from BY4743 S. cerevisiae after incubation in various reducing agents for 120 min. (B to G) Yeast incubated in reducing agents after 2 h have compromised plasma membrane PI fluorescent (bright red) yeast, and bright-field microscopy visualized the total amount of yeast (dark spots) after exposing yeast to reducing agents compared to yeast in carbonate buffer only (H). Values in panel A are single technical replicates and are representative of 2 experiments. Download FIG S2, PDF file, 2.8 MB. Copyright © 2020 Cohen et al. 2020 Cohen et al. This content is distributed under the terms of the Creative Commons Attribution 4.0 International license.

### Releasing active cell wall enzymes for *in vitro* analysis.

**FIG 6:**
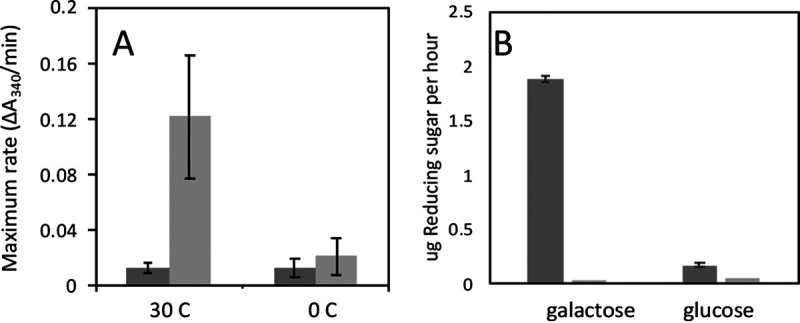
Release of cell wall enzymes from intact cells. Cells were incubated for 1 h in TEA buffer and then centrifuged, and enzyme activities were determined. (A) GAPDH release after incubation at 30°C or 0°C in the absence of DTT (dark gray) or with 100 μM DTT present (light gray). (B) Invertase activity after growth in galactose (to allow enzyme expression) or glucose (which represses expression). The substrate sucrose was added to the assay (left bars, dark gray) or omitted (right bars, light gray).

Since 100 μM DTT treatment did not compromise the plasma membrane, we wanted to know if we could use that concentration to extract cell wall proteins. Cells were incubated in 100 μM DTT for 60 min at 30°C. When we used a concentration of 2.5 × 10^6^ cells per ml (the concentration used during *in situ* cell surface GAPDH assays), there was negligible GAPDH activity released into the supernatant (not shown). However, at concentrations of 2 × 10^8^ cells per ml and above, we could monitor supernatant for NADH production. We estimate the GAPDH released by this method was less than 1% of the total GAPDH present in the wall, based on the level of activity associated with whole cells. This procedure released GAPDH when the cells were incubated at 30°C but not when they were incubated on ice (Fig. 6A). This method also released extracellular invertase from cells grown in galactose (Fig. 6B). Similar to the results for GAPDH, the amount of assayable invertase released was about 1% of the total invertase in the walls of intact cells. Therefore, extraction with 100 μM DTT can extract limited but assayable quantities of enzymes in cell walls.

To test other published cell wall extraction procedures, we treated cells with β1,3 glucanase, mild alkaline treatment, or reducing agents (5, 6, 42, 45). These methods failed to extract GAPDH without compromising the plasma membrane. Cell wall proteins extracted on ice in 100 mM Tris, pH 9.4, supplemented with 2% sorbitol excludes cytosolic proteins such as Cof1 (46). However, we were unable to detect GAPDH activity in these extracts (data not shown). Treating cells with Zymolyase, a lytic β1,3 glucanase, in the presence of 1 M sorbitol released GAPDH into the medium. However, some cells lysed rapidly and other cells appeared to be intact but stained with PI. Therefore, neither mild base extraction nor a spheroplast procedure specifically solubilized cell wall GAPDH.

To assess effects of mild base alone or with increased amounts of reductant, we suspended S. cerevisiae in 100 mM carbonate buffer containing several different concentrations of β-mercaptoethanol (βME), DTT, and TCEP for 2 h. Cells were pelleted by centrifugation, and supernatants were assayed for GAPDH activity *in vitro* while cells were stained with propidium iodide and visualized to monitor plasma membrane leakage. At high concentrations of βME or DTT, GAPDH was released, but a large proportion of yeast treated with these concentrations readily took up propidium iodide (47) (Fig. S2). Therefore, incubations in high concentrations of reducing agents probably released cytosolic proteins in addition to cell wall material.

## DISCUSSION

Our results point to several practical approaches to assay cell wall enzymes in yeast. We have screened assay procedures and found conditions that facilitate quantitative enzyme assays without compromising the integrity of the plasma membrane. Consequently, we can estimate minimum cell surface concentrations of GAPDH as the amount of active enzyme. Additionally, selective inactivation of GAPDH, coupled with kinetics of recovery of the activity, yielded a minimum estimate of the secretion rate. Thus, the results establish criteria for determination of concentrations and secretion rates for fungal cell wall enzymes.

### Cell wall GAPDH.

Enzymological data lead to estimates for the amount of active enzyme on each cell surface. GAPDH enzyme assays showed NADH reduction of about 0.25 *A*_340_ units per hour for 5 × 10^5^ cells. Because the molar extinction coefficient of NADH is 6.2 × 10^3^ M^−1 ^cm^−1^, this corresponds to production of about 3 × 10^−10 ^μmol of NADH per cell per min. Given the specific activity of yeast GAPDH, this amount of activity would result from about 4 × 10^4^ molecules of GAPDH per cell (38). This number is similar to that of other cell surface molecules, such as the S. cerevisiae sexual agglutinins (48). Note, however, that this concentration does not account for any surface GAPDH that is enzymatically inactive. For comparison, invertase, a conventionally secreted highly expressed surface enzyme, is about 100-fold higher in fully derepressed cells (49). Therefore, cell surface GAPDH concentrations are commensurate with its frequent detection in wall proteomics studies but are significantly lower than maximal levels of a highly expressed surface enzyme.

### Biotinylation as a tool for selectively deactivating GAPDH.

Biotinylation is frequently used as a mechanism of tagging cell wall proteins for Western blot analysis or proteomics (50), including unconventionally secreted proteins such as enolase (51) and the Hsp70 members Ssa1 and Ssa2 (52, 53). To our knowledge, it has not been used to deactivate enzymes *in situ*. Biotinylation ablated GAPDH activity but did not alter external invertase activity, so not all external enzymes can be deactivated in this manner. Therefore, labeling intact cells with sulfo-NHS-LC biotin did not globally alter classical secretion and is minimally invasive. Sulfo-NHS-LC biotin contains a charged sulfonate group, making it membrane impermeant (44). Therefore, the reagent specifically deactivated GAPDH that was external to the membrane. Propidium iodide staining and flow cytometry experiments demonstrated that the plasma membrane remained intact as GAPDH activity returned to the surface within 30 to 60 min (Fig. 3 and 5). Therefore, we conclude that cell surface GAPDH is specifically inactivated by sulfo-NHS-LC biotin and that plasma membrane remains intact both after inactivation and during extended incubations in assay buffer.

**FIG 7:**
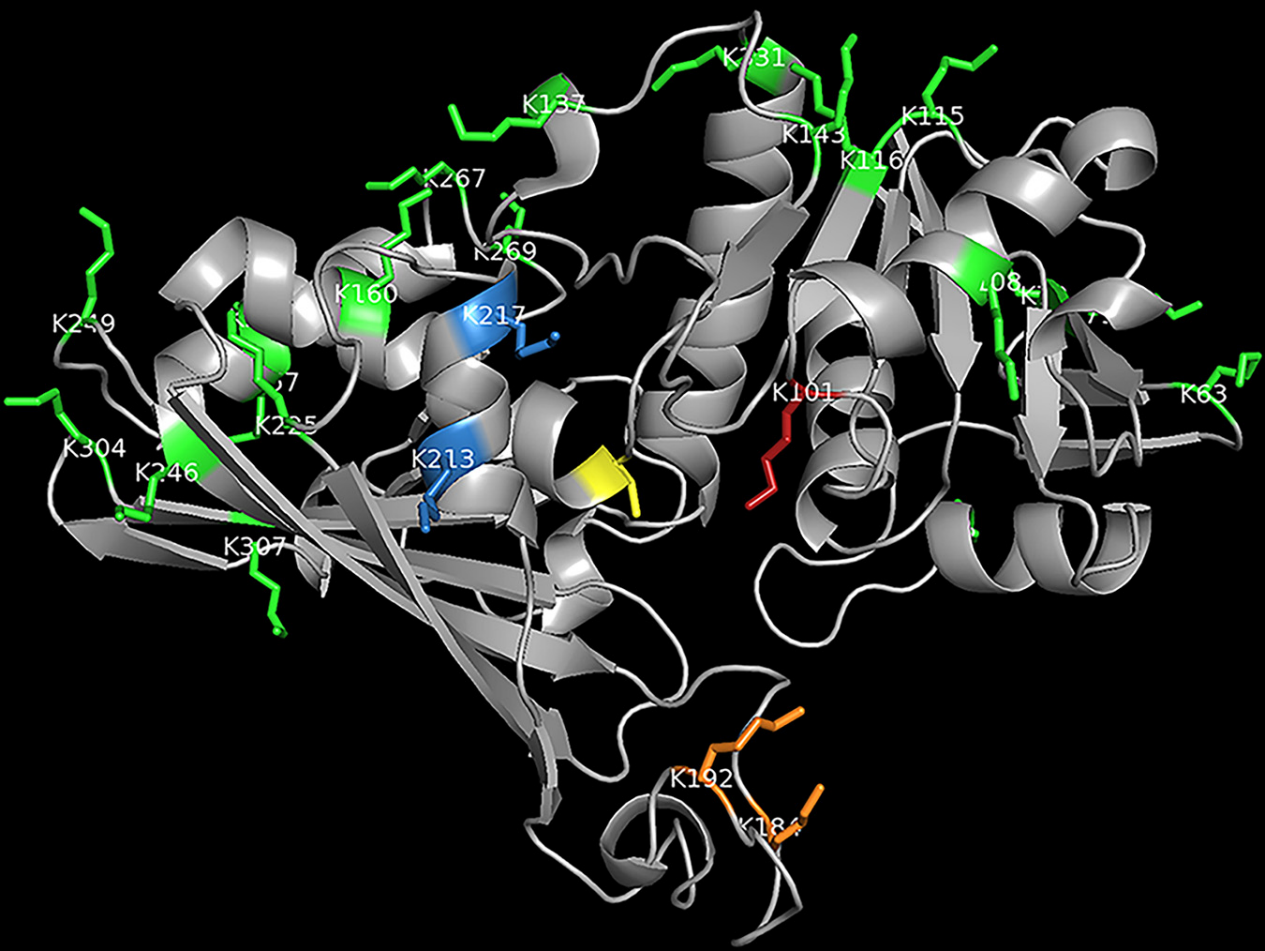
Ribbon diagram of yeast GAPDH (PDB entry 4IQ8). Active-site residue Cys150 is colored yellow. All Lys residues are labeled and their sidechains rendered. Lys residues are color-coded by straight-line distance from active-site residue Cys150 (yellow): red, ∼13 Å; blue and orange, ∼18 Å; green, >20 Å.

Sulfo-NHS-LC biotin reacts with primary amines. Based on the structure of yeast Tdh3, which is reported to be the major form of GAPDH in the cell wall with Tdh2 during exponential growth phase (1), we identified lysine residues near the catalytic cysteine, the glyceraldehyde-3-phosphate binding domain, and the NAD binding domain (54). There are no Lys residues in the active site. However, five Lys residues are within 20 Å of the active-site residue Cys150, close enough that the 22-Å-long biotin moiety could reach (Fig. 7). Among these, Lys184 and Lys 192 (orange) are in the NAD^+^-binding loop, and it is likely that biotinylation would disrupt NAD^+^ binding, electron transfer, and/or quaternary structure (55). Therefore, it is likely that sulfo-NHS-LC is directly inactivating GAPDH by covalently modifying lysines near its active site.

### Secretion of GAPDH.

After inactivation of surface GAPDH, steady-state levels of activity were reestablished in the wall within an hour of incubation at 30°C (Fig. 3). Thus, the rate of GAPDH secretion was about 4 × 10^4^ molecules of active enzyme per hour under these conditions. The recovery was dependent on incubation temperature, implying that the recovery was due to a secretory event rather than passive leakage from the cytosol through damaged membranes.

### Reducing agents can compromise the plasma membrane.

One striking observation we made is that the millimolar concentrations of reducing agents used to extract cell wall proteins (5, 30, 56, 57) can compromise the plasma membrane, leading to propidium iodide uptake. This is consistent with observations of Curwin et al., who used cofilin as a marker for cytosolic leakage (46).

To extract cell wall proteins for enzymology while avoiding cytosolic contamination, we recommend incubating S. cerevisiae in 100 μM DTT as described above and monitoring yeast for cytosolic permeability with propidium iodide. GAPDH and invertase are considered periplasmic (held in place between the wall and the plasma membrane) (1, 58, 59), so this technique can extract proteins associated with the innermost layer of the cell wall. We also recommend passing supernatant through a 0.22-μm filter to avoid contaminating extracts with unpelleted cells. Unfortunately, extraction with 100 μM DTT is inefficient based on the observation that about 1% of the activity of GAPDH or invertase is released into the medium.

Thus, there are techniques for assaying cell wall enzymes *in situ.* A minimum estimate is 4 × 10^4^ molecules of GAPDH in the wall of each cell in exponentially growing cultures. After inactivation of wall-resident enzyme, this same amount can be secreted in an hour. We conclude that enzymatic assays are suitable for studying unconventional secretion and speculate that these techniques will be useful for other cell wall proteins (20).

## MATERIALS AND METHODS

### Determining GAPDH activity at the cell surface.

S. cerevisiae strain BY4743 was grown in yeast extract-peptone-dextrose (YPD) to log phase, pelleted, and resuspended in either 20 mM sodium citrate buffer, pH 5, or Tris-buffered saline (TBS), pH 7, to an OD_600_ of 1.25. At this concentration, 20 μl contained 5 × 10^5^ cells. Twenty microliters of cells from each concentration was loaded into a microcentrifuge tube and placed on ice. To initiate the reaction, 180 μl of TEA buffer (40 mM triethanolamine [Sigma], 50 mM Na_2_HPO_4_, 7.5 mM EDTA, pH 8.6) containing 100 μM DTT, 1 mM NAD^+^ (Alfa Aesar), and 7 μl of 100 mg/ml glyceraldehyde-3-phosphate (Cayman Scientific or Sigma-Aldrich) from frozen stocks was used. The cell suspension was incubated at 30°C for 30 min and placed on ice for 5 min to retard the reaction, and then S. cerevisiae was pelleted by centrifuging at full speed (13,000 × *g*) for 1 min. Supernatant (180 μl) was collected, and the *A*_340_ was measured on a Biotek Synergy 2 plate reader. Supernatant (180 μl) from a negative-control reaction of 5 × 10^5^ cells without glyceraldehyde-3-phosphate or without cells was used as a blank. To determine kinetics of NADH production, cells were incubated for 0 to 120 min before analysis of supernatants.

To determine how incubation in DTT alters GAPDH activity on the surface over time, 500,000 yeast cells in 20 μl of TBS were mixed with 160 μl of TEA buffer with or without DTT (100 μM) and incubated for the times stated. After incubation, NAD and glyceraldehyde-3-phosphate were added, and 2 μl of 10 mM DTT was added to reaction mixtures lacking DTT. The tubes were incubated at 30°C for 30 min, and the supernatant was collected and analyzed for NADH production by reading the *A*_340_.

### Extraction of cytoplasmic GAPDH.

S. cerevisiae cells were lysed with glass beads in PBS with a 1:1,000 dilution of yeast protease inhibitor cocktail set IV (Calbiochem), the lysate was cleared by centrifugation at 4°C at full speed on a microcentrifuge, and supernatant was analyzed for GAPDH activity.

### *In vitro* GAPDH kinetics.

Ten-microliter volumes of either a cell wall extract, whole-cell lysate, or 10-fold dilutions were loaded into a microplate. A BioTek synergy 2 plate reader was prewarmed to 30°C, 90 μl of TEA buffer containing 1 mM NAD^+^, glyceraldehyde 3 phosphate, and 100 μM DTT was added, and the OD_340_ was monitored over 60 min. Negative-control wells contained 10 μl of the buffer used to extract protein mixed with the other reagents, or extract was mixed with TEA buffer containing all of the reagents except for glyceraldehyde-3-phosphate. To calculate GAPDH activity, we determined the slope of the steepest linear part of the OD_340_ curve during the first 5 to 60 min.

### Biotinylation of GAPDH.

Cytosolic lysate was covalently modified with or without 1 mg/ml sulfo-NHS-LC biotin (ApexBio) for 1 h. The biotinylated and nonbiotinylated lysates were then washed in a 10-kDa membrane cutoff filter (Sigma) with PBS, and 10 μl was loaded into a microplate with 90 μl substrate and analyzed for GAPDH activity.

To biotinylate intact yeast, S. cerevisiae cells were washed and resuspended at an OD_600_ of 2.5 to 5 in PBS with or without 1 mg/ml sulfo-NHS-LC biotin for 1 h at 4°C or on ice. The treated cells were washed twice and resuspended in TBS to measure GAPDH activity as described above or in citrate buffer to measure invertase.

### Whole-cell invertase assays.

S. cerevisiae BY4741 and BY4743 were grown to an OD_600_ of 0.45 to 0.55 in yeast extract-peptone medium with 2% galactose as the carbon source (YPGal) and concentrated to an OD_600_ of 1 in 20 mM sodium citrate buffer (pH 5). One hundred fifty microliters of this cell suspension was mixed with 50 μl of 0.4 M sucrose to a final OD_600_ of 0.75 with 0.1 M sucrose and incubated at 30°C. After half-hour suspensions were pelleted, reducing sugar released was quantified by boiling a 1:1,000 dilution in tetrazolium blue (Sigma) for 3 min, and the OD_670_ was measured in either a Spectronic 600 or BioTek Synergy plate reader. The OD_670_ was used to quantify reducing sugar against a set of glucose standards (60). All assays presented were carried out in duplicate and are representative of 3 or more independent experiments.

To measure invertase extracted from cell walls, S. cerevisiae was grown to an OD_600_ of 0.5 in YPD (to suppress invertase) or YPGal (to derepress invertase expression) and resuspended to an of OD_600_ of 20 and 23, respectively, in TEA buffer (40 mM triethanolamine [Sigma], 50 mM Na_2_HPO_4_, 7.5 mM EDTA, pH 8.6) containing 100 μM DTT for 60 min at 30°C. One hundred fifty microliters of 1,000 × *g* supernatant was collected and mixed with 50 μl of sucrose in citrate buffer as stated above, except reactions were run for 60 min and the reaction was terminated by immediately diluting in tetrazolium blue, which is prepared in NaOH and will denature enzyme. Micrograms of reducing sugar released by invertase was measured at the *A*_670_ and compared to a glucose curve (60).

### Propidium iodide staining.

S. cerevisiae cells were treated as stated, stained with either 2 to 20 μg/ml of propidium iodide (PI) (Sigma), with concentrations within ranges reported for live/dead staining (4, 61, 62), and visualized under fluorescence microscopy using a tetramethyl rhodamine isocyanate filter.

### Flow cytometry.

We incubated BY4743 at a concentration of 2.5 × 10^5^ per ml in TEA buffer (pH 8.6) with 0 to 10 mM DTT at 30°C for 0 to 90 min, and at each time point we removed 100 μl, added PI to a final concentration of 2 μg/ml, incubated samples for an additional 5 min to ensure all dead cells take up the dye (62), and measured PI fluorescence on a BD Accuri flow cytometer.

### Cell wall extraction procedures.

To generate spheroplasts, S. cerevisiae strain BY4743 was resuspended in PBS with or without 1 M sorbitol. One unit of Zymolyase (Zymogen) was added to the mixture, and lysis was monitored visually in the tube lacking sorbitol. Spheroplasted yeasts were identified using phase-contrast microscopy at ×400 magnification. The spheroplasts stabilized in sorbitol were pelleted at 2,000 rpm, and supernatant was collected and assayed for enzyme activity *in vitro.* The spheroplasts were washed in PBS plus 1 M sorbitol and stained with PI as described above (the volume of PI added did not exceed 1% of the total volume). Reducing agents for GAPDH release and cell viability were added to 2 × 10^6^ cells/ml in 100 mM carbonate buffer containing the indicated concentrations of reducing agents at 30°C for 2 h. An aliquot of cells was stained with PI as described above, remaining cells were pelleted, and 10 μl of serial dilutions was used to measure GAPDH activity in the supernatant.

To extract cell wall proteins using 100 μM DTT, S. cerevisiae cells were washed 2× in TEA buffer and concentrated to an OD_600_ of 10 to 30. DTT was added to a final concentration of 100 μM from a 100 mM frozen stock solution, and the cells were incubated either on ice or at 30°C for 60 min and then pelleted. Ninety percent of the supernatant was collected to avoid disturbing the pellet. In later experiments the supernatant was passed through a 0.22-μm Durapore filter (Sigma) to remove any remaining cells.
