# Supplementary figures and images for: Enzymatic Analysis of Yeast Cell Wall-Resident GAPDH and Its Secretion
Source: mSphere. 2020 Dec 16;5(6):e01027-20. doi: 10.1128/mSphere.01027-20 (PMC7771233; doi:10.1128/mSphere.01027-20)

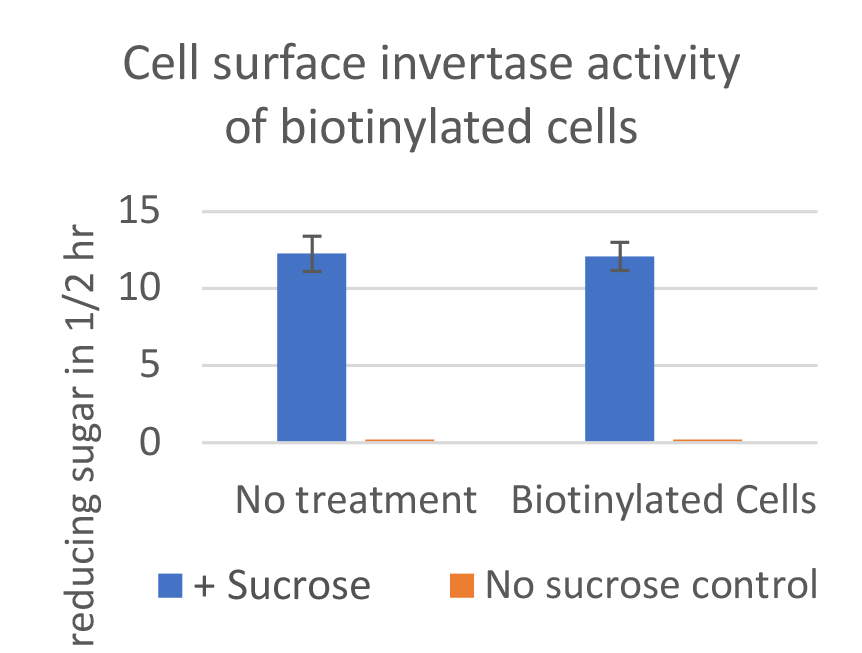

Supplement: FIG S1 [file mSphere.01027-20-sf001.tif]

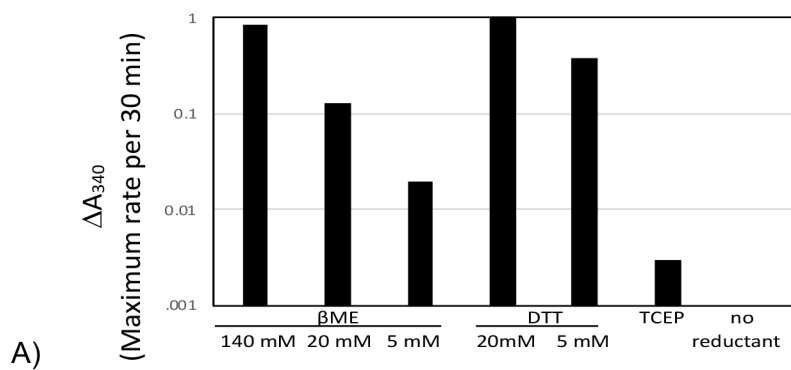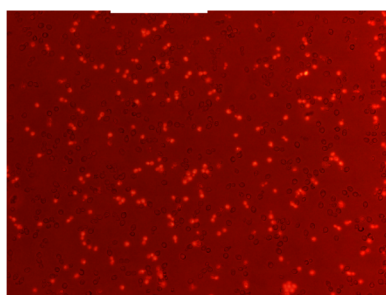

B) 140 mM βME (1%)

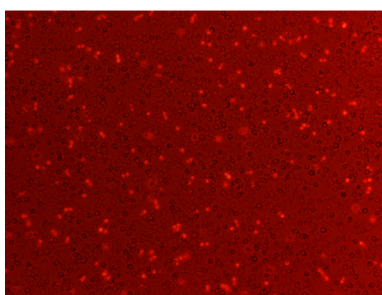

C) 20mM βME

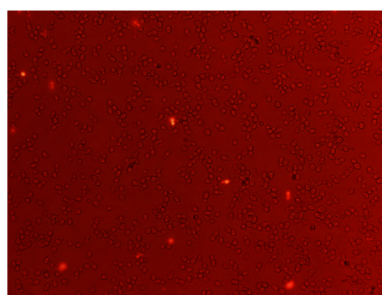

D) 5mM βME

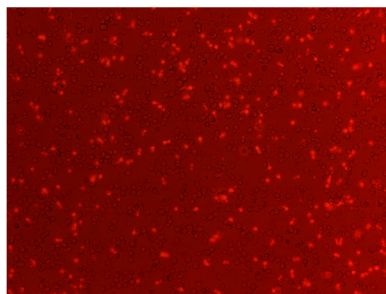

E) 20 mM DTT

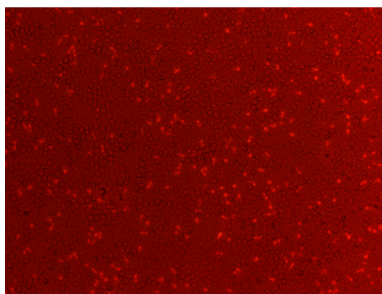

F) 5mM DTT

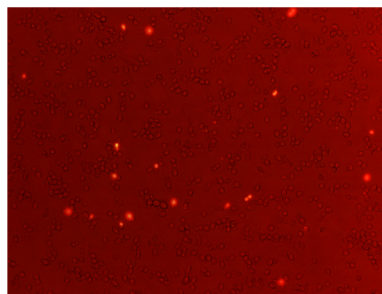

G) 5mM TCEP

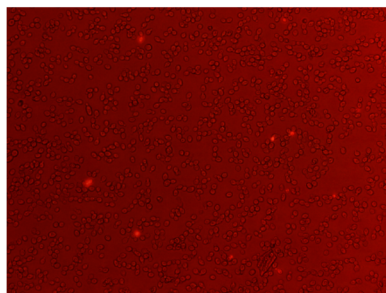

H) No reducing agent

Supplement: FIG S2 [file mSphere.01027-20-sf002.pdf]
